# miRNA-204-5p acts as tumor suppressor to influence the invasion and migration of astrocytoma by targeting ezrin and is downregulated by DNA methylation

**DOI:** 10.1080/21655979.2021.2000244

**Published:** 2021-11-24

**Authors:** Haibo Jiang, Ruixiang Ge, Siwen Chen, Laiquan Huang, Jie Mao, Lili Sheng

**Affiliations:** a Department of Emergency Intensive Care Unit, Yijishan Hospital, First Affiliated Hospital of Wannan Medical College, Wuhu City, China; b Department of Neurosurgery, Yijishan Hospital, First Affiliated Hospital of Wannan Medical College, Wuhu City, China; c Department of Reproductive Medicine, Yijishan Hospital, First Affiliated Hospital of Wannan Medical College, Wuhu City, China; d Department of Hematology, Yijishan Hospital, First Affiliated Hospital of Wannan Medical College, Wuhu City, China; e Department of Neurosurgery, Shenzhen Hospital of Southern Medical University, Shenzhen City, China; f Department of Oncology, Yijishan Hospital, First Affiliated Hospital of Wannan Medical College, Wuhu City, China

**Keywords:** miRNA-204-5p, astrocytoma, DNA methylation, ezrin, invasion and migration

## Abstract

microRNAs (miRNAs), through their regulation of the expression and activity of numerous proteins, are involved in almost all cellular processes. As a consequence, dysregulation of miRNA expression is closely associated with the development and progression of cancers. Recently, DNA methylation has been shown to play a key role in miRNA expression dysregulation in tumors. miRNA-204-5p commonly acts in the suppression of oncogenes in tumors. In this study, the levels of miRNA-204-5p were found to be down-regulated in the astrocytoma samples. miRNA-204-5p expression was also down-regulated in two astrocytoma cell lines (U87MG and LN382). Examination of online databases showed that the miRNA-204-5p promoter regions exist in CpG islands, which might be subjected to differential methylation. Subsequently, we showed that the miRNA-204-5p promoter region was hypermethylated in the astrocytoma tissue samples and cell lines. Then we found that ezrin expression was down-regulated with an increase in miRNA-204-5p expression in LN382 and U87MG cells after 5-aza-2ʹ-deoxycytidine (5ʹAZA) treatment compared with control DMSO treatment. In addition, LN382 and U87MG cells treated with 5ʹAZA exhibited significantly inhibited cell invasion and migration . In a recovery experiment, cell invasion and migration returned to normal levels as miRNA-204-5p and ezrin levels were restored. Overall, our study suggests that miRNA-204-5p acts as a tumor suppressor to influence astrocytoma invasion and migration by targeting ezrin and that miRNA-204-5p expression is downregulated by DNA methylation. This study provides a new potential strategy for astrocytoma treatment.

miRNA-204-5p act as a tumor suppressor in astrocytomas. miRNA-204-5p promoter regions exist within a hypermethylation state. DNA methylation-induced changes in the expression of miRNA-204-5p have a direct effect on the invasion and migration of human astrocytoma cells and that ezrin may be a potential target involved in these effects.

## 1. Introduction

Astrocytoma is a type of glioma that arises from the neuroectodermal tissue. It is the most common primary brain tumor, characterized by rapid growth and highly aggressive malignant development [1]. The prognosis of astrocytoma is closely related to the World Health Organization (WHO) classification of the tumor. The most common histological subtype is glioblastoma (GBM), which is a highly heterogeneous and aggressive malignancy. With surgical interventions and chemotherapy, the median survival time is approximately 12–15 months [2–5]. To date, very few chemotherapeutic agents have been effective against astrocytoma, and only three (carmustine wafers, temozolomide and bevacizumab) have been used in clinical treatment [6]. DNA methylation and microRNA (miRNA) regulation are intensive areas of research as they have been implicated in the disease development [7] and consequently might provide insight into the discovery of novel therapeutic agents.

DNA methylation is an important epigenetic process that regulates gene expression and occurs mainly within CpG islands in promoter regions and in the vicinity of the transcription start site. Promoter CpG island methylation is often associated with changes in gene expression. In addition, DNA methylation can control gene expression by altering the chromosome structure and the conformation of DNA, leading to changes in the stability of DNA and DNA-protein interactions, etc [8]. As a consequence, malignant tumors are often characterized by abnormal levels of DNA methylation.

In recent years, DNA methylation has been reported to play a key regulatory role in the dysregulation of miRNA expression in tumors [9]. miRNAs are small, single-stranded, noncoding RNAs, which participate in post-transcriptional regulation, mostly through binding to the 3ʹ untranslated region (3ʹ-UTR) of their target gene transcript [10,11]. In mammals, miRNAs affect the activity of approximately 2/3 of all protein-coding genes. They target key signaling pathways and are involved in all esssential cellular processes, such as apoptosis, cell differentiation [12]. Therefore, disordered expressions of miRNAs are closely related to the initiation and development of cancers. A previous study has shown that miRNA-204-5p acts as a tumor suppressor and its dysregulation is closely related to the initiation and development of astrocytoma [13,14]. Ezrin is involved in multiple cellular functions including cell adhesion, migration, and cell surface structures organization. We have shown in our previous work that miRNA-204-5p inhibits astrocytoma cell migration and invasion and functions as a direct negative regulator of *EZRIN* gene expression [15]. However, the specific mechanism connecting miRNA-204-5p and ezrin is still unknown, making it unclear whether an additional mechanism of action causes tumors to occur. We speculate that miRNA-204-5p promoter methylation may be relevant to astrocytoma cell migration and invasion by affecting the expression levels of miRNA-204-5p and ezrin.

In this study, we aimed to discovery new therapeutic agents for astrocytoma. By adjusting the DNA methylation level of the miRNA-204-5p promoter region, we showed that DNA methylation plays a key regulatory role by which miRNA-204-5p inhibits the invasion and migration of astrocytoma cells by inhibiting the expression of ezrin. Thus, miRNA-204-5p and ezrin might serve as targets to suppress astrocytoma recurrence and metastasis.

## 2. Materials and methods

### 2.1. Cell lines and cell culture

Primary normal human astrocytes (NHAs) and two different malignant human astrocytoma cell lines, U87MG and LN382, were purchased from American Type Culture Collection (Manassas, VA). The cell lines were cultured according to the manufacturer’s recommendations in Dulbecco’s modified Eagle’s medium (DMEM) supplemented with 10% fetal bovine serum (Gibco) in a humidified incubator at 37°C with a 5% CO_2_ atmosphere.

### 2.2. Patients

A total of 30 astrocytoma tissue samples and ten normal brain tissue samples were obtained from patients who underwent surgery at the Department of Neurosurgery, Yijishan Hospital of Wannan Medical College. The tumor tissue samples were collected from patients who had no prior radiotherapy or chemotherapy, and the normal brain tissue samples had no detectable lesions. All specimens were obtained with informed consents from the patient or their guardians and the study was approved by the Clinical Research Ethics Committee of Wannan Medical College (Approval No.AECYJS-2015010).

### 2.3. *RNA extraction, reverse transcription and qRT-PCR* [15]

Total RNA was extracted from cell lines and tissue samples by using TRIzol reagent (Sangon Biotech) according to the manufacturer’s protocols. For miRNA quantification, Bulge-loop^TM^ miRNA qRT-PCR Primer Sets (one RT primer and a pair of qRT-PCR primers for each set) for miRNA-204-5p were designed by RiboBio (Guangzhou, China). Relative expression levels were normalized against U6 small nuclear RNA levels. The mRNA expression levels of ezrin were analyzed by SYBR Green qRT-PCR, and GAPDH was used for normalization. Total RNA from samples was first reverse-transcribed using the Thermo ScientificTM RevertAidTM First Strand cDNA Synthesis Kit. The qRT-PCR primers used in the study were as follows: Ezrin, forward, 5ʹ-GCTGGATAAGAAGGTGTCTGC-3ʹ and reverse, 5ʹ-GGTCCCTGGTAAGTTTGTGC-3ʹ; GAPDH, forward 5ʹ-GGAGCGAGATCCCTCCAAAAT-3ʹ and reverse, 5ʹ-GGCTGTTGTCATACTTCTCATGG-3ʹ; U6 forward 5ʹCTCGCTTCGGCAGCACA-3ʹ and reverse, 5ʹ-AACGCTTCACGAATTTGCGT-3ʹ. The manufacturer of primers are Sangon Biotech(Shanghai, China). The reaction system was as follows: 10 μl SYBR, 0.4 μl snRNA specific Primer set, 0.2 μl Taq DNA polymerase, 2 μl miRNA RT product, 7.4 μl Sterilized water. The PCR program was as follows: sample denaturing at 95°C for 3 min; 40 cycles of denaturing at 95°C for 12s, annealing at 62°C for 40s. We used denaturing agarose gel electrophoresisto to assess RNA and cDNA quality. qRT-PCR was performed by using a Bio-Rad CFX96 Fast Real-Time PCR system. We used the 2^−ΔΔCT^ method to calculate the relative expression levels of ezrin and miRNA-204-5p.

### 2.4. *DNA isolation, bisulfite modification and methylation-specific PCR* [9]

Genomic DNA was extracted with the TIANGEN DNA Kit (Beijing), and all steps were carried out in accordance with the manufacturer’s instructions. Total DNA was stored in a − 80°C freezer to prevent DNA degradation until use. Bisulfite conversion of genomic DNA was performed using an EZ DNA Methylation-Gold™ kit, and all steps were carried out in accordance with the manufacturer’s instructions. Methylation-specific PCR (MSP) was performed with bisulfite-modified DNA as the template. The reaction system was as follows: 2 μl DNA, 1 μl primer, 12.5 μl Taq Mix, and 8.5 μl RNase-free water. The PCR program was as follows: sample denaturing at 94°C for 5 min; 40 cycles of denaturing at 94°C for 30 s, annealing at 57°C for 30 s, and extension at 72°C for 30 s; and elongation at 72°C for 7 min. The specific MSP primers for miRNA-204-5p were as follows: Methylated – forward, 5ʹ-AAGATGTTTTGTGTAAGAAAGTGTAAGTT-3ʹ; Methylated- reverse, 5ʹ-CGCGCTCCCTCTCCCG-3ʹ; Unmethylated- forward, 5ʹ-AAGATGTTTTGTGTAAGAAAGTGTAAGTT-3ʹ; Unmethylated- reverse, 5ʹ-CAAAATACACACTCCCTCTCCCA-3ʹ. MSP end products were amplified by agarose gel electrophoresis.

### 2.5. *Bisulfite genomic sequencing* [9]

We used NCBI (National Center of Biotechnology Information) to detect miRNA-204-5p’s location in the human genome. We used the online databases EMBL-EBI (European Molecular Biology Laboratory’s European Bioinformatics Institute) and MethPrimer to analyes that the miRNA-204 promoter regions exist in CpG islands.Primers were designed with MethPrimer. The specific bisulfite genomic sequencing PCR (BSP) primers for miRNA-204-5p were as follows: forward, 5ʹ-AGGATGAAGATTGTTAATTAGGGT-3ʹ and reverse, 5ʹ-AAATCAAAACAACCTACTCCAAAA-3ʹ. The total length of the ?predicted product was 371 bp #determined by what?, which included 11 CpG sites. The PCR program was as follows: sample denaturing at 94°C for 5 min; 40 cycles of denaturing at 94°C for 30 s, annealing at 57°C for 30 s, and extension at 72°C for 30 s; and elongation at 72°C for 7 min. Quantity One software was used to analyze BSP products. The purification and recovery of DNA were performed by using TIANGEN kits (Beijing). All sequencing steps were performed in a centralized lab (Sangon Biotech Company, Shanghai).

### 2.6. *5-Aza-2ʹ-deoxycytidine treatment* [16]

U87MG and LN382 cells were treated with 5-aza-2ʹ-deoxycytidine (5ʹAZA; Sigma-Aldrich, St. Louis, MO, USA). When U87MG cells and LN382 cells grew to 70% confluency, they were treated with 5 µM 5ʹAZA for 72 hours, and the cell culture medium was replaced every 24 hours. Following treatment, RNA, DNA and protein were extracted for detecting the relative expression levels of miRNA-204-5p and ezrin.

### 2.7. *Transient transfection* [17]

Cells were plated in a 6-well plate and cultured until approximately 90% confluent. We transfected the cell lines with miRNA-204-5p mimics and inhibitors, as well as negative control (NC) mimics and inhibitors (RiboBio, Guangzhou, China) by using Lipofectamine 3000 (Company) according to the manufacturer’s instructions. The transfection efficiencies were checked by using qRT-PCR.

### 2.8. *Real-time cell analyzer (RTCA) migration and invasion assays* [18]

RTCA technology integrates microelectric sensors into the underside of a migration or invasion plate, and the resistance of the underside changes when cells grow on the underside. Changes in resistance are recorded by the sensor and are automatically calculated by RTCA software to generate a cell index that reflects the number of cells on the underside. Real-time and dynamic quantitative analysis of cells was used to track cell migration and invasion. This experiment used the xCELLigence RTCA DP (model: 3 × 16) instrument to record and analyze data. The CIM-Plate 16 board was used for migration and invasion. Prior to the RTCA experiment, the whole machine was placed in a 5% CO2, 37°C cell incubator, and the data were collected only when the temperature of the RTCA instrument was consistent with that of the incubator. The RTCA results were cell index curves, and the Mann-Kendall trend test was used to determine whether there was a significant difference. The following experiments were performed in triplicates. For the RTCA migration assay, 100 μl of serum-free medium suspension to each well of the upper chamber of the CIM-Plate 16 board and the cell concentration was set to 20,000. 10% serum was added to the lower chamber and the plate was put into the RTCA instrument. After 0.5 hours (when the CIM-Plate 16 board was heated to 37°C), the RTCA program was started. After the RTCA experiment was terminated at 24 hours, the data were statistically analyzed. For an RTCA invasion assay, 30 µl of diluted matrix gel was used to coat each well before cell seeding. The remaining steps were the same as those described in the RTCA migration assay, except the number of seeded cells was changed to 40,000 cells per well.

### 2.9. Statistical analysis

Experimental data are presented as the mean ± SEM. Human tissue samples statistical analyses were performed using Paired *t* tests. Cell lines statistical analyses were performed using unpaired t tests. Data normality distribution were performed using kolmogorov-smirnov test. (SPSS 16.0, SPSS Inc., Chicago). Differences with P < 0.05 were considered to be statistically significant. The test method for the RTCA curve was adopted from the Mann-Kendall trend test: ① Calculated difference sequence: A and B with two RTCA curves, where A_i_ and B_i_ indicate A and B i duration curves corresponding to cell indices, respectively; the difference sequence is defined as D_j_ = abs(A_j_-B_j_), where abs represents absolute value operations, and ② Mann-Kendall trend test for sequence D. We used R languages to perform statistical analysis.

## 3. Results

Our previous studies indicated that miRNA-204-5p was a direct negative regulator of ezrin, which was one of its downstream targets. In this study, we want to show that DNA methylation plays a key regulatory role by which miRNA-204-5p inhibits the invasion and migration of astrocytoma cells by inhibiting the expression of ezrin. The levels of miRNA-204-5p in 30 freshly dissected astrocytoma and ten normal brain tissue samples were determined by qRT-PCR, subsequently, by performing bisulfite genomic sequencing PCR (BSP) and methylation-specific PCR (MSP) in 5 astrocytoma and 2 normal brain tissue samples. Tumor cell’s invasion, and migration were measured by Real-time Cell Analyzer (RTCA) migration and invasion assays.

### 3.1. miRNA-204-5p expression was downregulated in human astrocytoma tissue samples and cell lines

**Figure 1.**
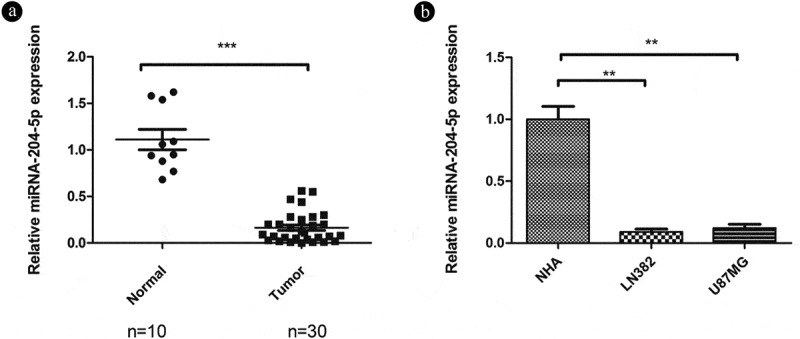
Relative expression of miRNA-205-5p in tissue samples and cell lines. (a) miRNA-204-5p levels in 30 freshly dissected astrocytoma samples and 10 normal brain tissue samples, as determined by qRT-PCR. The expression of miRNA-204-5p was obviously downregulated in the astrocytoma tissue samples compared with the normal brain tissue samples. ***P < 0.001. (b) miRNA-204-5p levels in normal human astrocytes (NHAs) and 2 astrocytoma cell lines, as determined by qRT-PCR. The expression of miRNA-204-5p was obviously downregulated in the 2 astrocytoma cell lines compared with the NHAs. **P < 0.01. Data are expressed as the mean ± SD of three independent experiments

As miRNA-204-5p often has been implicated in the inhibition of oncogenes and likely participates in the initiation and development of human cancers [19], we aimed to explore the role of miRNA-204-5p in astrocytoma. qRT-PCR was used to detect relative expression in 30 freshly dissected astrocytoma samples and 10 normal brain tissue samples. The expression of miRNA-204-5p was markedly downregulated in the astrocytoma tissue samples compared with the normal brain tissue samples (Figure 1(a)). We also compared miRNA-204-5p expression in two different astrocytoma and NHA control cell lines. Similarly, the two astrocytoma cell lines also showed downregulated expression of miRNA-204-5p compared with the NHAs (Figure 1(b)), consistent with the tumor suppressive role of miRNA-204-5p in astrocytoma.

### 3.2. miRNA-204-5p acts as a tumor suppressor by inhibiting cell migration and invasion in astrocytoma cells

**Figure 2.**
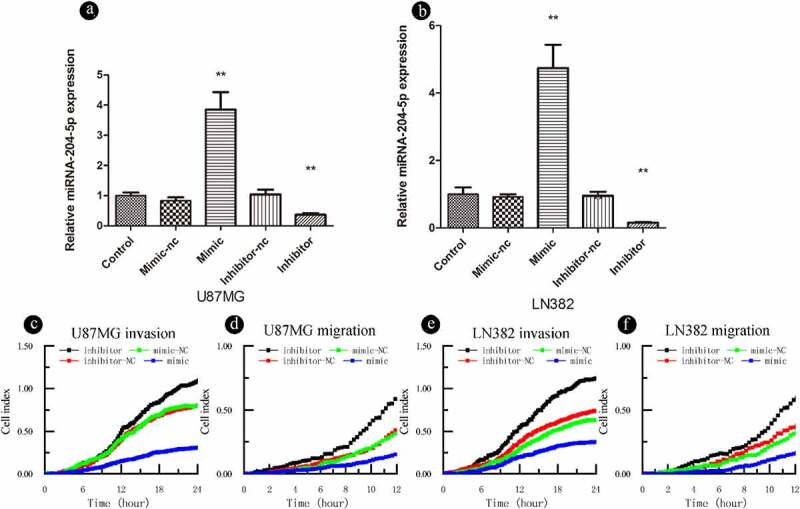
miRNA-204-5p acts as a tumor suppressor gene and inhibits the invasion and migration of astrocytoma cells. (a, b) The transfection efficiency of miRNA-204-5p was determined. (C, D, E, F) Invasion and migration could be inhibited by upregulating miRNA-204-5p expression in LN382 and U87MG cells. Invasion and migration could be promoted by knocking down miRNA-204-5p expression in LN382 and U87MG cells

To further explore the biological roles of miRNA-204-5p in astrocytoma, we investigated its effects on astrocytoma cell migration and invasion. The expression of miRNA-204-5p was modulated by using a miRNA-204-5p mimic and miRNA-204-5p inhibitors (Figure 2(a,b))). RTCA invasion and migration assays indicated that increased expression of miRNA-204-5p by the miRNA mimic significantly inhibited cell invasion (Figure 2(c)) and migration (Figure 2(d)) in LN382 and U87MG cells compared to negative control treatment. On the other hand, when miRNA-204-5p was inhibited, cell invasion (Figure 2(e)) and migration (figure 2(f)) were increased. These results suggest that miRNA-204-5p acts as a tumor suppressor by inhibiting cell migration and invasion in astrocytoma cells.

### 3.3. Identification of hypermethylated CpG islands in miRNA-204-5p in astrocytomas

**Figure 3.**
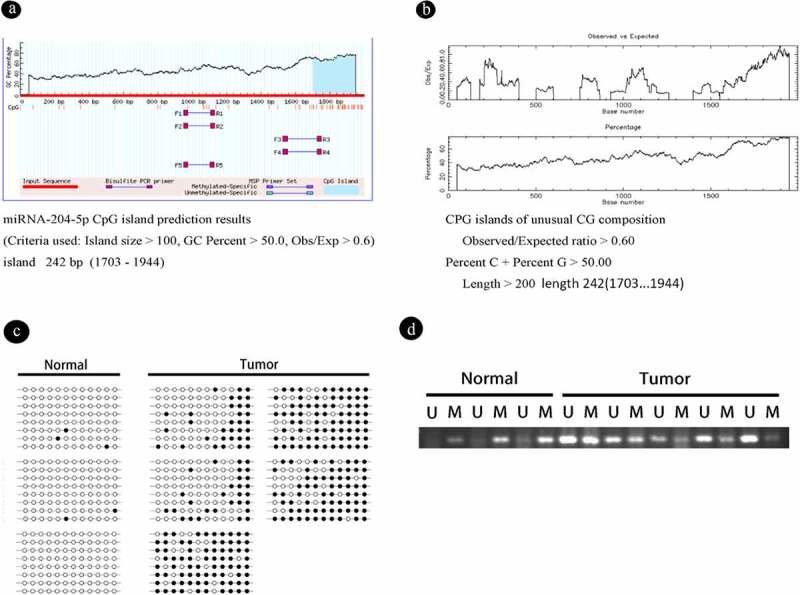
DNA methylation is present in astrocytoma tissue samples and cell lines, and the degree of methylation can be reduced by using 5-aza-2ʹ-deoxycytidine (5ʹAZA). (a, b) We used NCBI to detect miRNA-204-5p in the middle of TRPM3 and used the online databases EMBL-EBI and MethPrimer – to suggest that the miRNA-204-5p promoter regions exist in CpG islands, which is located in the area 1703–1944, and the promoter length is 242 bp. (c) The methylation statuses of 5 astrocytoma and 3 normal brain tissue samples were determined by bisulfite genomic sequencing PCR (BSP). Black dots represent methylated CpGs, and white dots represent unmethylated CpGs. The columns represent CpG dinucleotides 1–12, and the rows represent clones 1–8. (d) The miRNA-204-5p methylation statuses of 5 astrocytoma and 3 normal brain tissue samples were determined by MSP. M: PCR products specifically amplified by MSP for methylated DNA. U: PCR products specifically amplified by MSP for unmethylated DNA

It has been reported that miRNA-204-5p is located between exons 7 and 8 of the *TRPM3* gene (REF). In NHAs and astrocytoma cell lines, the expression of miRNA-204-5p was related to that of *TRPM3*, suggesting that miRNA-204-5p and *TRPM3* might be subjected to similar regulatory mechanisms. We first analyzed the sequence of miRNA-204-5p with BLAST (NCBI), and found that it was indeed located in *TRPM3* with an E value of 0 has relatively high credibility. Next, we examined the online databases EMBL – EBI and MethPrimer predictions to show that the miRNA-204-5p promoter regions exist within CpG islands that is located in the area 1703–1944, and the promoter length is 242 bp (Figure 3(a,b)). We used BSP to verify this prediction in six astrocytoma and two normal brain tissue samples.

The BSP results showed that the promoter CpG islands were mostly unmethylated in NHAs with relatively high levels of miRNA-204-5p expression and in non-neoplastic brain tissues. In contrast, the promoter CpG islands of miRNA-204-5p were hypermethylated in most astrocytoma specimens, including astrocytoma cells with downregulated miRNA-204-5p expression and astrocytoma tumor samples, suggesting an essential role for promoter methylation in miRNA-204-5p downregulation (Figure 3(c)). Similar results were obtained by using the MSP method (Figure 3(d)).

### 3.4. DNA methylation can directly regulate the expression of miRNA-204-5p in astrocytoma cell lines

**Figure 4.**
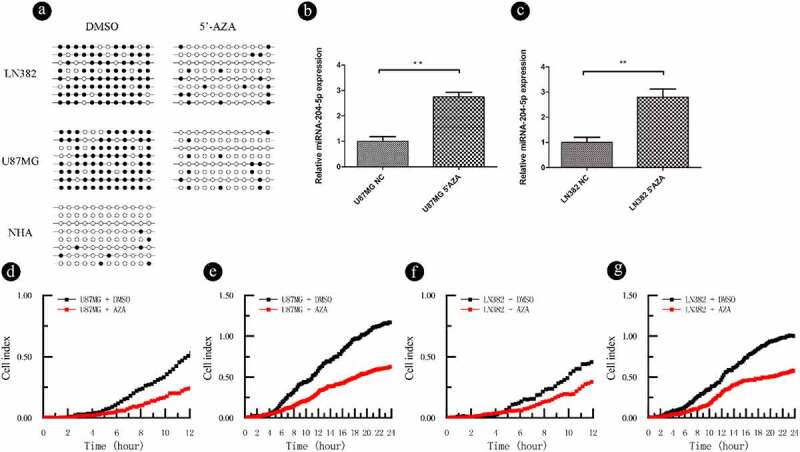
DNA methylation can regulate the expression of miRNA-204-5p in astrocytoma cell lines and affect the invasion and migration of these cell lines. (a) The methylation statuses of U87MG and LN382 cells were determined by BSP before and after 5ʹAZA treatment. Black dots represent methylated CpGs, and white dots represent unmethylated CpGs. The columns represent CpG dinucleotides 1–12, and the rows represent clones 1–8. (b, c) The expression of miRNA-204-5p was upregulated in LN382 and U87MG cells after 5ʹAZA treatment compared with control DMSO treatment. **P < 0.01. Data are expressed as the mean ± SD of three independent experiments. (D, E, F, G) LN382 and U87MG cells treated with 5ʹAZA had significantly inhibited cell invasion and migration compared to control DMSO-treated cells

**Figure 5.**
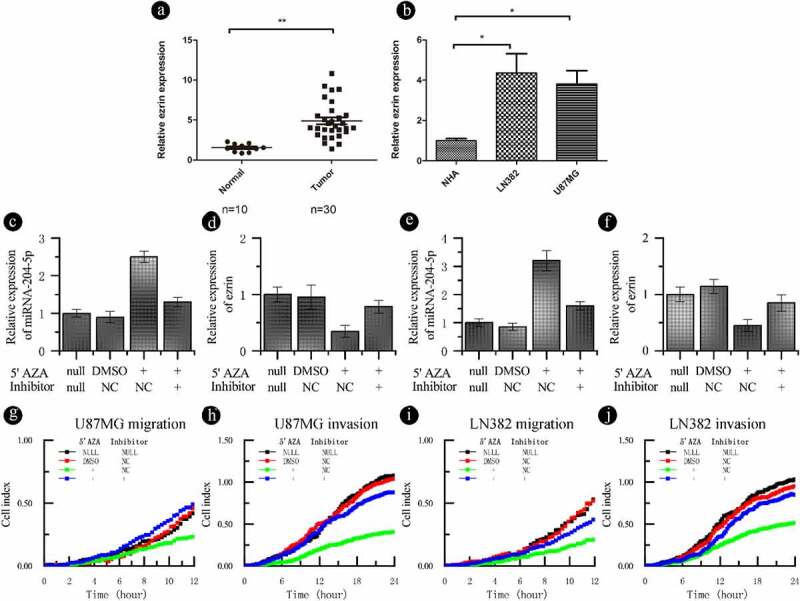
Ezrin expression is downregulated by miRNA-204-5p and regulated by DNA methylation. (a) The ezrin levels in 30 freshly dissected astrocytoma samples and 10 normal brain tissue samples were determined by qRT-PCR. The expression of ezrin was obviously upregulated in the astrocytoma tissue samples compared with the normal brain tissue samples. **P < 0.01. (b) The expression of ezrin was obviously downregulated in NHAs compared with 2 astrocytoma cell lines. *P < 0.05. Data are expressed as the mean ± SD of three independent experiments. (C, D, E, F) The expression of ezrin in a recovery experiment, in which we treated U87MG and LN382 cells with a miRNA-204-5p inhibitor +5ʹAZA, was measured. The relative expression of ezrin and miRNA-204-5p returned to near normal. **P < 0.01. Data are expressed as the mean ± SD of three independent experiments. (G, H, I, J) Cell invasion and migration were evaluated in the recovery experiment. When we treated U87MG and LN382 cells with the miRNA-204-5p inhibitor +5ʹAZA, cell invasion and migration returned to near normal levels

The aforementioned results indicate that miRNA-204-5p expression is associated with DNA methylation. To test if the expression of miRNA-204-5p is under the direct regulation of promoter methylation, two astrocytoma cell lines (U87MG and LN382) were treated with the demethyltransferase inhibitor 5ʹAZA. We showed that the miRNA-204-5p promoter? was demethylated following 5ʹAZA treatment in U87MG and LN382 cells by using BSP (Figure 4(a)). In addition, qPCR analysis showed that the expression of miRNA-204-5p was upregulated in LN382 and U87MG cells after 5ʹAZA treatment compared with control DMSO treatment (Figure 4(b,c)). RTCA invasion and migration assays indicated that compared with control DMSO treatment, treatment with 5ʹAZA significantly inhibited cell invasion (Figure 4(d,e)) and migration (figure 4(f,g)) in LN382 and U87MG cells. When we treated U87MG and LN382 cells with the miRNA-204-5p inhibitor and 5ʹAZA, the relative expression of miRNA-204-5p returned to near normal levels (Figure 5(c,e)). These data support a direct role of miRNA-204-5p expression in astrocytoma cell invasion and migration, which was regulated by differential methylation.

### 3.5. Ezrin expression is upregulated in astrocytoma and varies with miRNA-204-5p methylation

Previously, our group has demonstrated an inverse correlation between miRNA-204-5p and ezrin expression in glima cells [15]. In this study, we used qRT-PCR to detect the relative expression of ezrin in correlation with miRNA-204-5p in our samples. As expected, higher expression levels of ezrin was detected in the astrocytoma tissue samples (Figure 5(a)) and astrocytoma cell lines (Figure 5(b)) with low miRNA-204-5p expressions compared with their respective controls. As the miRNA-204-5p expression in the U87MG and LN382 cell lines increased after 5ʹAZA treatment, ezrin expression decreased, which was rescued by the use of miRNA-204-5p inhibitor in 5ʹAZA-treated cells (Figure 5(d,f)). Meanwhile, the relative expression changes of miRNA-204-5p and ezrin correlated with differences in cell migration and invasion (Figure 5(g-j)).

## 4. Discussion

Astrocytoma is the most prevalent malignancy in the brain, with high morbidity and mortality rates. GBM is one of the deadliest forms of astrocytoma and is characterized by a high recurrence rate post-surgery, resulting in a poor prognosis. The median patient survival time is less than 15 months after diagnosis [20,21]. Postoperative chemotherapy with temozolomide (TMZ) can improve the median patient survival time. However, approximately half of the patients with malignant astrocytoma are resistance to TMZ, which is one of the main causes of GBM chemotherapy failure [22]. To effectively combat the progression of astrocytoma, new therapeutic strategies must be adopted to combat migratory apoptosis-resistant and proliferative astrocytoma cells [23].

miRNAs are involved in virtually all cellular processes [12,24,25] and may serve as new molecular targets for astrocytoma treatment [26,27]. Previous studies have shown that miRNA-204-5p plays important tumor suppressive roles in cancers, including breast cancer [28,29], endometrial carcinoma [30], pancreatic cancer [31], gastric cancer [32,33], renal clear cell carcinoma [17,34], and head and neck cancer [35]. Studies in NSCLC suggested that downregulation of miRNA-204-5p and direct upregulation of its target genes, as well as activation of TFs [7,36–39] are associated with tumor progression and drug resistance [40]. Similar reports suggest that miRNA-204-5p promotes apoptosis in prostate cancer cells by targeting Bcl2 [36]. Our previous study has shown that the downregulation of miRNA-204-5p expression in astrocytoma impairs its inhibitory effects on astrocytoma cell migration and invasion [15]. Consistent with an immunosuppressive role of miRNA-204-5p, our current study also found lower expression of miRNA-204-5p in astrocytoma tissues and cell lines compared with normal samples. By using specific mimics and inhibitors, our findings also demonstrated a direct role of miRNA-204-5p in suppressing cellular invasion and migration. These results confirmed the tumor-suppressive function of miRNA-204-5p in astrocytoma.

In this study, we set out to further investigate the mechanism(s) underlying miRNA-204-5p function. miRNAs can be regulated by epigenetic modifications such as DNA methylation [7,36,37]. Aberrant DNA methylation is an important indicator of the inactivation of tumor suppressor genes [38]. CpG islands from the promoter of miRNAs are the target sequences for hypermethylation or hypomethylation in multiple cancers [39]. Xia *et al*. [41] has demonstrated that downregulation of miRNA-204 is associated with the methylation of its promoter’s CpG sites in papillary thyroid carcinoma (PCT). In line with their study, we found the presence of CpG islands in the promoter of miRNA-204-5p in astrocytoma. We further showed promoter hypermethylation and downregulated expression of miRNA-204-5p in both astrocytoma tissues and cell lines compared with normal samples. Moreover, we investigated the connections between the methylation of miRNA-204-5p promoter and miRNA-204-5p target gene ezrin. High expression of ezrin significantly affects the prognosis of astrocytoma patients [42]. Our previous studies indicated that miRNA-204-5p was a direct negative regulator of ezrin, which was one of its downstream targets [15]. Treatment with 5ʹAZA confirmed that the expression of miRNA-204-5p was indeed modulated by promoter methylation, and consequently the expression of its direct target, ezrin, as well as its effect on cell invasion and migration. miRNA inhibitors blocked the influence of miRNA-204-5p on ezrin expression and recovered cell invasion and migration.

Our findings may have important implications in the development of new therapeutic options for astrocytoma patients. For example, miRNA-based anti-cancer therapies, such as miRNA reduction or inhibition and miRNA replacement or restoration [41,43], might be an approach to block tumor progression [24–27,44–47]. Recently, it has been shown that PEGylated polymer nanoparticles loaded with miRNA-204-5p can be used to treat colon cancer [48]. In recognition of their functional importance, additional epigenetic modulators, such as DNA methyltransferase inhibitors and histone deacetylase inhibitors, have been developed to enable restoration of expressions and functions of inappropriately silenced genes in cancers [49,50]. While clinical efficacies of these epigenetic modulators have been demonstrated in cancer patients [51], their clinical applications are often limited due to relatively high toxicity as result of the lack of specificity across the entire genome [16,52]. Similarly, each miRNA can bind to many target genes and each gene can be regulated by multiple miRNAs. Here, we showed that miRNA-204-5p plays a vital role in suppressing tumor invasion and migration and ezrin is one of its direct downstream genes in astrocytoma. Future studies should explore the additional downstream targets of miRNA-204-5p, as well as the mechanisms by which ezrin regulates cell invasion and migration.

## 5. Conclusion

In summary, this study demonstrates that DNA methylation-induced changes in the expression of miRNA-204-5p have a direct effect on the invasion and migration of human astrocytoma cells and that ezrin may be a potential target involved in these effects. Results from our study may greatly facilitate the development of potential therapeutic targets for astrocytoma patients, and further investigations on the roles of ezrin, miRNA-204-5p, and DNA methylation in astrocytoma are needed.
